# Adipose Tissue Dysfunctions in Response to an Obesogenic Diet Are Reduced in Mice after Transgenerational Supplementation with Omega 3 Fatty Acids

**DOI:** 10.3390/metabo11120838

**Published:** 2021-12-04

**Authors:** Alexandre Pinel, Jean-Paul Rigaudière, Béatrice Morio, Frédéric Capel

**Affiliations:** 1 Unité de Nutrition Humaine (UNH), Faculté de Médecine, Institut National de Recherche Pour L’Agriculture, L’Alimentation et L’Environnement (INRAE), Université Clermont Auvergne, 28 Place Henri Dunant, 63001 Clermont-Ferrand, France; alexandre.pinel@uca.fr (A.P.); jean-paul.rigaudiere@inrae.fr (J.-P.R.); 2 CarMeN Laboratory, INSERM U1060, INRAE U1397, University Lyon 1, 69310 Pierre Bénite, France; beatrice.morio@inrae.fr

**Keywords:** obesity, adipose tissue, metabolism, gene expression, mitochondria

## Abstract

Obesity is characterized by profound alterations in adipose tissue (AT) biology, leading to whole body metabolic disturbances such as insulin resistance and cardiovascular diseases. These alterations are related to the development of a local inflammation, fibrosis, hypertrophy of adipocytes, and dysregulation in energy homeostasis, notably in visceral adipose tissue (VAT). Omega 3 (n-3) fatty acids (FA) have been described to possess beneficial effects against obesity-related disorders, including in the AT; however, the long-term effect across generations remains unknown. The current study was conducted to identify if supplementation with n-3 polyunsaturated FA (PUFA) for three generations could protect from the consequences of an obesogenic diet in VAT. Young mice from the third generation of a lineage receiving a daily supplementation (1% of the diet) with fish oil rich in eicosapentaenoic acid (EPA) or an isocaloric amount of sunflower oil, were fed a high-fat, high-sugar content diet for 4 months. We explore the transcriptomic adaptations in each lineage using DNA microarray in VAT and bioinformatic exploration of biological regulations using online databases. Transgenerational intake of EPA led to a reduced activation of inflammatory processes, perturbation in metabolic homeostasis, cholesterol metabolism, and mitochondrial functions in response to the obesogenic diet as compared to control mice from a control lineage. This suggests that the continuous intake of long chain n-3 PUFA could be preventive in situations of oversupply of energy-dense, nutrient-poor foods.

## 1. Introduction

Obesity is associated with a higher risk of disability and co-morbidities, such as cardiovascular diseases (CVD), type 2 diabetes, and cancer. Childhood obesity predisposes to the same disorders at a higher risk [1,2]. Obesity is a multifactorial process, involving genetic, environmental, and behavioral factors. Imbalance in the energy intake and expenditure is a major contributor to fat mass accretion. White adipose tissue (WAT) is the major site of energy storage but it also has an endocrine function by the secretion of adipokines that contribute to nutrient metabolism and insulin sensitivity in peripheral tissues [3,4]. Alteration of WAT physiology during obesity leads to insulin resistance, low grade inflammation, perturbation of fuel selection by metabolic tissues, contributing to a global alteration of energy homeostasis. These abnormalities are crucial factors of the increased risk for disability and mortality and the strong socioeconomic impact of obesity [5]. Most healthy and preventive lifestyle recommendations aim at increase physical activity, and reduce energy intake by eating a diet rich in fruits and vegetables and poor in sugars. Such a diet should provide natural bioactive compounds with antioxidant and anti-inflammatory properties [6]. Furthermore, recommendations for the fatty acid (FA) composition of dietary fats emphasize on the limitation of saturated FA (SFA), an increase in omega 3 (n-3) polyunsaturated FA (PUFA), and a controlled omega 6 (n-6) PUFA intake in order to reach an n-6:n-3 ratio close to 2:1, contrasting with the value of 15–20:1 observed in a typical Western diet [7,8,9]. N-3 PUFAs are bioactive nutrients with protective roles against endothelial dysfunctions, inflammation, heart disease, and obesity [10,11]. By contrast, n-6 PUFA have pro-inflammatory and pro-adipogenic effects [12].

N-3 PUFA must be supplied by the diet, notably α-linoleic acid (ALA 18:3 n-3), found in plants and which is converted by the body to eicosapentaenoic acid (EPA, 20:5 n-3) and docosapentaenoic acid (DHA, 22:6 n-3). EPA and DHA are mainly found in seafood and were demonstrated to be the most bioactive FA of the family in their native or oxidized form [13,14,15]. Finally, docosapentaenoic acid (DPA, 22:5 n-3) is an intermediate of EPA and DHA, which may also have metabolic and healthy properties [16]. It has been observed that supplementation of an obesogenic diet with EPA induced an accumulation of DPA and DHA in different organs and limited fat mass gain, inflammation, and metabolic abnormalities in mice [17,18]. The mechanisms remain partially described but could involve an anti-lipogenic effect of EPA [19,20]. Evidence was also provided about the improvement of adipose tissue secretory profile and inflammation in rodents receiving a high-fat diet supplemented with n-3 PUFA [21,22]. Recently, our group has demonstrated that dietary supplementation of three generations of mice with EPA could alleviate the deleterious metabolic impact of an obesogenic diet [23] in the third generation (F3 mice). Transgenerational supplementation with n-6 or n-3 PUFA could induce specific biological adaptations, which could affect fatty acid metabolism and susceptibility to fat mass accretion [12]. We observed that transgenerational supplementation with fish oil (1% of the diet), rich in EPA, reduced the activation of fibrotic processes in skeletal muscle and contributed to a better metabolic flexibility in the liver in F3 mice exposed to an obesogenic diet through several molecular adaptations [23,24], but the consequences on the AT remained to be explored. As the AT dysfunctions are a key element in the progression of obesity-related comorbidities, nutritional strategies using n-3 PUFA, and aiming at the improvement of its secretory and metabolic activities, were proposed [25].The aim of the present study was to explore how the transgenerational supplementation with n-3 PUFA, mainly in the form of EPA, affected adipose tissue biology and adaptation after an obesogenic challenge.

## 2. Results

### 2.1. High-Fat Diet Related Transcriptomic Adaptation in VAT Are Reduced after Transgenerational Intake of EPA

The details of the breeding strategy to obtain three generations of mice receiving a supplementation with EPA and of the obesogenic challenge could be found in Section 4.1. We previously described that the biometric and metabolic consequences of 17 weeks of an obesogenic diet differed between F3 mice from the EPA lineage (HFepa) compared to a control/oleic lineage (HFoleic) [23]. Briefly, HFepa mice gained less weight, fat mass, and exhibited better metabolic function compared to HFoleic mice and to a reference group of F3 mice from the control lineage/oleic ( group “Ref”) receiving a normocaloric diet. Adipose tissue samples from these animals were used for mRNA isolation for a transcriptomic exploration. Three pairwise comparisons were performed. HFoleic and HFepa groups were compared to Ref group, and a comparison between HFoleic and HFepa groups was also performed.

**Figure 1:**
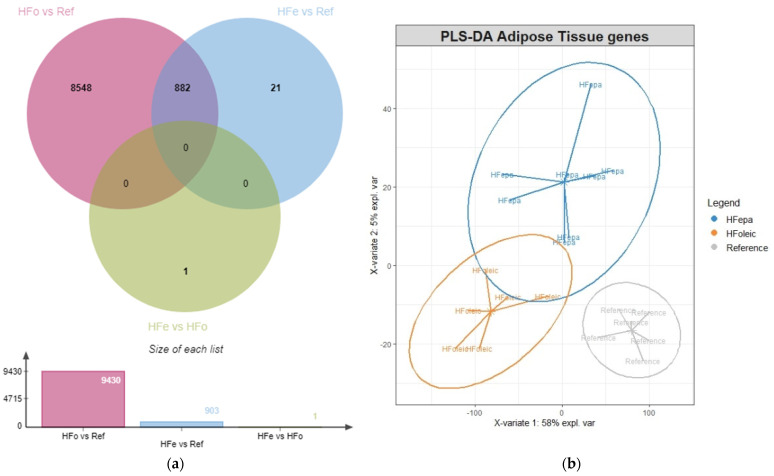

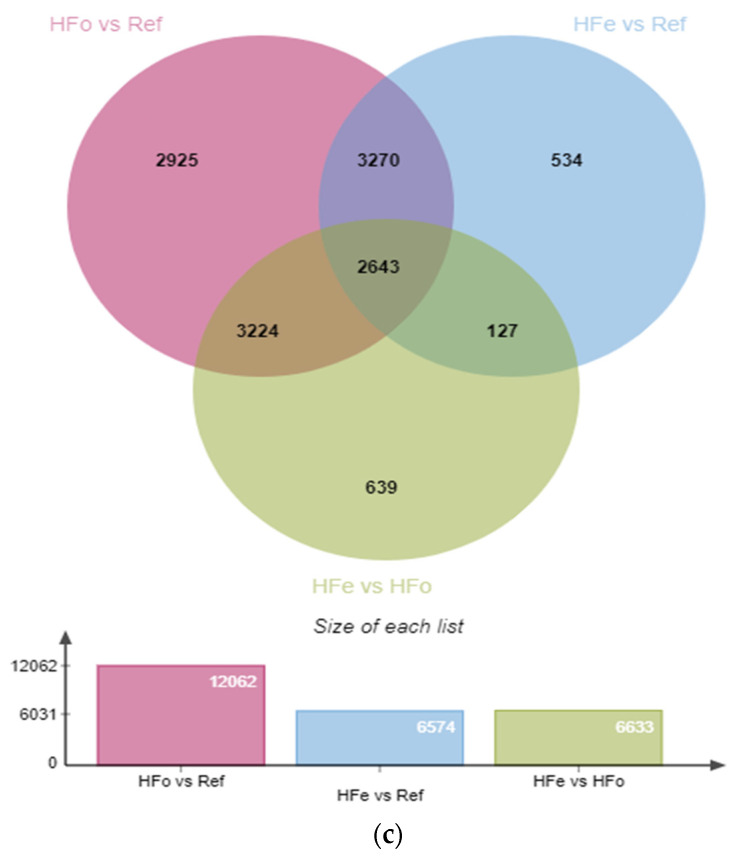
Modulation of gene expression adaptation during an obesogenic diet following transgenerational intake of EPA. (**a**) Venn diagram illustrating the genes differentially expressed in each comparison (significantly identified mRNA with *p* < 0.05 after FDR correction for multiple testing). (**b**) PLS-DA plot of mRNA expression levels of significantly regulated transcripts (*p* < 0.05 after correction for multiple testing). (**c**) Venn diagram illustrating the genes differentially expressed in each comparison (significantly identified mRNA with *p* < 0.05 before FDR correction for multiple testing). HFoleic (or HFo), mice from oleic/control lineage fed with the obesogenic diet; HFepa (or HFe), mice from EPA/omega 3 lineage fed with the obesogenic diet; Reference (or ref), mice from oleic/control lineage fed with the normocaloric diet.

Out of the 38,181 probes exhibiting a valid signal, 12,319 were differentially expressed between HFoleic and reference groups (9430 unique transcripts, 4876 over represented in HFoleic, and 4554 over represented in reference) when the Benjamini–Hochberg procedure was used for multiple testing correction. Using the same criteria, the number of significantly regulated transcripts was lower between the HFepa and reference groups, 1026 were differentially expressed (903 unique genes, 532 over represented in HFepa, and 371 over represented in reference). Among the significantly regulated genes identified in the comparisons of HFoleic and HFepa against the reference group, 882 regulations were common among them (Figure 1a). One transcript was differentially expressed between HFepa and HFoleic using the multiple testing correction. All the differential expressions that were also evaluated by RT-qPCR were validated (Table 1). Expression data of all these transcripts were used for a PLS-DA, showing a clear separation of the three groups, and illustrating the differential impact of the high-fat diet in HFepa group compared to HFoleic group (Figure 1b).

### 2.2. Comparison of Cell Type Distribution in VAT

**Figure 2:**
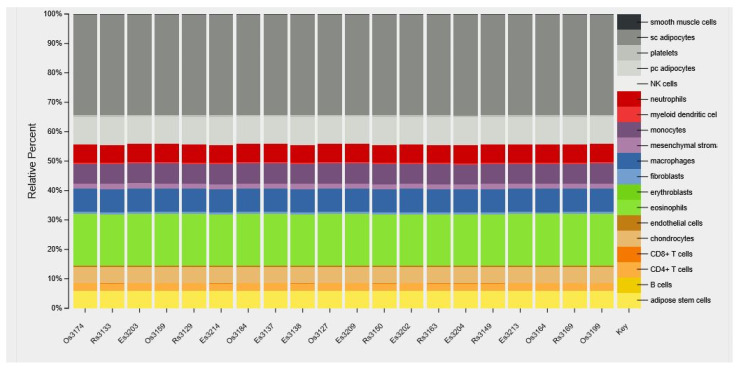
Cell-type heterogeneity in the different adipose tissue samples. CIBERSORT was used for the measurement of cell-type proportions from adipose-tissue microarray samples. Sample numbers starting with O, E, and R are from HFoleic, HFepa, and Reference groups, respectively. HFoleic, mice from oleic/control lineage fed with the obesogenic diet; HFepa, mice from EPA/omega 3 lineage fed with the obesogenic diet; Reference, mice from oleic/control lineage fed with the normocaloric diet.

The effect of the intervention on VAT cell-type composition was explored using CIBERSORT. According to the in silico strategy developed with this online software [26], the gene expression data were used to estimate the relative abundance of the different cells types found in adipose tissue. As illustrated in Figure 2, the representation of the main cell types did not differ between samples suggesting that neither the obesogenic challenge, nor the previous transgenerational supplementation with n-3 PUFA, had an effect on the proportion of adipocytes (43%), immune cells (41%), adipose stem cells (6%), and chondrocytes (5.5%).

### 2.3. Transgenerational Supplementation with EPA-Induced Differential Regulation of Biological Pathways and Processes in VAT

Gene Set Enrichment Analysis (GSEA) was used to explore how the transgenerational intake of omega 3 FA impacted the regulation of biological processes and pathways induced by the obesogenic challenge. GSEA applies a statistical analysis to identify concordant variation in expression value of set of genes between two biological states with adjustment for multiple testing. Consequently, we explored the regulation of biological processes and pathways using the different databases implemented in GSEA pathways (WIKI, KEGG, Biocarta, and Reactome) on all differentially expressed mRNA in the three pairwise comparisons: HFoleic vs. Ref (unique transcripts), HFepa vs. Ref (6574 unique transcripts), and HFepa vs. HFoleic (6633 unique transcripts) according to the statistical analysis of microarray data before FDR adjustment as described in Section 4.4. In these conditions, the representation of the numbers of regulated transcripts in each comparison showed that 5867 out of the 12,062 transcripts differentially regulated between HFoleic and Ref groups also exhibited a differential expression level between HFepa and HFoleic groups (Figure 1c). Measurements of mRNA levels by RT-qPCR were performed on a subset of genes and validated the differential expression between groups (Table 1), notably between HFoleic and HFepa.

**Figure 3:**
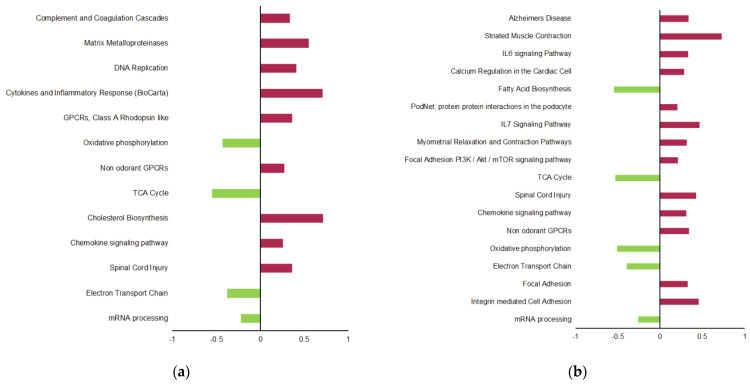

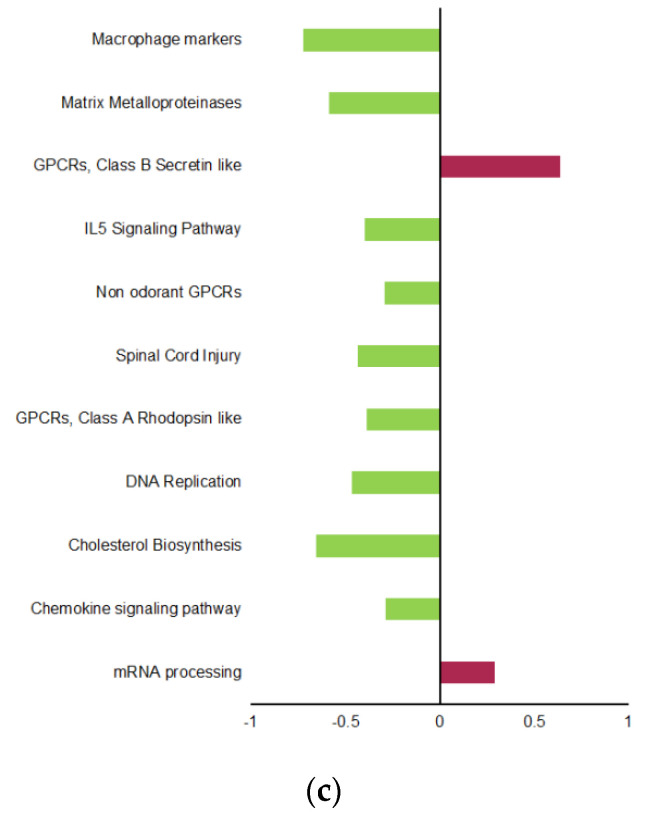
Regulation of pathways in VAT according to Gene Set Enrichment Analysis in the different pairwise comparisons and the WIKI database. Left panel illustrates significantly regulated pathways in HFoleic group compared to the Reference (**a**). Central panel illustrates significantly regulated pathways in HFepa group compared to the Reference (**b**). Right panel illustrates significantly regulated pathways in HFoleic group compared to HFepa (**c**). Red color means overrepresentation, green color means lower representation in HFoleic (compared to Ref in (**a**), compared to HFoleic in (**c**)) or HFepa (compared to Ref in (**b**)). HFoleic, mice from oleic/control lineage fed with the obesogenic diet; HFepa, mice from EPA/omega 3 lineage fed with the obesogenic diet; Reference, mice from oleic/control lineage fed with the normocaloric diet.

The exploration using GSEA of regulated processes and reactions showed that the obesogenic diet induced in HFoleic group a depletion in electron transport chain (ETC), oxidative phosphorylation genes, and enrichment in genes related to inflammation, cholesterol synthesis, extracellular matrix remodeling, and eicosanoid metabolism as compared to the Ref group (Figure 3a). Some genes from some of these pathways were selected for RT-qPCR validation analyses, such as *Stat3*, *Foxo3* (inflammation), and *Mmp13* (matrix remodeling).

In the HFepa group, the obesogenic diet also repressed gene sets related to ETC, oxidative phosphorylation, but increased the expression of gene sets involved in the PI3K-AKT-mTOR-signaling pathway, in addition to some sets related to inflammation compared to the Ref group (Figure 3b).

The numbers of genes related to the sets exhibiting a significant enrichment in both HFepa and HFoleic groups compared to the Ref group was clearly lower in the HFepa group (Figure 1c). GSEA identified significant changes between the HFepa and HFoleic groups (a complete list of differential processes and reactions is provided in Supplementary Table S1). Depletion in genes related to eicosanoid metabolism, chemokine signaling, macrophage markers, and cholesterol synthesis were observed in the HFepa group compared to the HFoleic group (Figure 3c). Trends were also observed for a differential regulation of the adipogenesis process (not shown), involving notably mRNA encoding leptin and Irs2, which exhibited a differential expression between the HFoleic and HFepa groups (Table 1).

A representation of the expression ratio between HFepa and HFoleic groups on the Chemokine signaling pathway showed an overexpression of *Akt* and *Pi3k* genes, and a downregulation of *Foxo* and *Gsk3* beta genes in the HFepa group (Supplementary Figure S1). The lower expression of *Foxo3* gene in HFepa group compared to both HFoleic and Ref groups was confirmed by RT-qPCR analysis (Table 1).

As illustrated in Figure 3c, only few pathways were overrepresented in HFepa group compared to HFoleic group. It concerned mRNA processing and G protein-coupled receptors (GPCRs). Many reactions extracted from the GeneTrail’s reactome database, which were depleted in HFepa group, concerned extracellular matrix, and concerned genes such as Mmp13 and Timp3.

**Figure 4:**
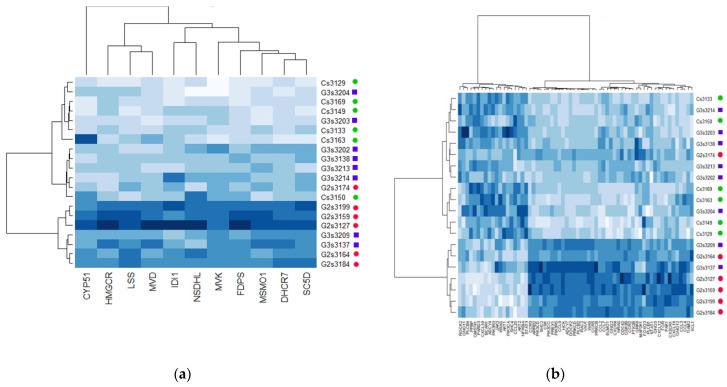
Heatmap displaying the expression level of genes related to cholesterol synthesis (**a**) and chemokine signaling (**b**) in VAT of the 3 groups of mice. Samples from Ref, HFoleic, and HFepa groups are illustrated with green circles, red circles and blue squares, respectively. Heatmap were generated using the correlation-based distance calculation and the ward method as clustering criteria. The magnitude of expression level is represented from white (low) to clear blue (intermediate) and dark blue (high). HFoleic, mice from oleic/control lineage fed with the obesogenic diet; HFepa, mice from EPA/omega 3 lineage fed with the obesogenic diet; Ref, mice from oleic/control lineage fed with the normocaloric diet.

The heatmap representation of correlation between expression data related to the genes involved in cholesterol metabolism and chemokine signaling showed a good separation of the animals according to their respective group (Figure 4). Of note, samples from HFepa group exhibited an expression pattern that was more similar to the Reference group.

### 2.4. Differential Regulation of Mitochondrial Processes in VAT between EPA and Control Lineages

As the obesogenic diet induced an enrichment in several pathways related to mitochondrial function or processes, we further extracted information using the MitoMiner database of mitochondrial targeted genes. We independently used the list of up and down regulated transcripts identified at *p* < 0.05 after FDR correction, for the HFoleic vs. Ref and HFepa vs. Ref comparisons. In agreement with the authors of MitoMiner [27], we extracted the list of genes related to mitochondria with a IMPI score confidence > 0.8. As compared to reference group, we identified 317 up- and 383 down-regulated genes in HFoleic, 28 up- and 42 down-regulated genes in HFepa (Supplementary Table S2). Details about the roles of these genes were obtained by an analysis of the related ontology to identify over represented KEGG pathways (Supplementary Table S3). It showed that the branched chain amino acid (BCAA) degradation pathway was commonly regulated in HFoleic and HFepa groups. A strong regulation of genes related to fatty acid oxidation (up or down regulated), glycolysis-related genes and a repression of oxidative phosphorylation pathway in HFoleic group only.

**Figure 5:**
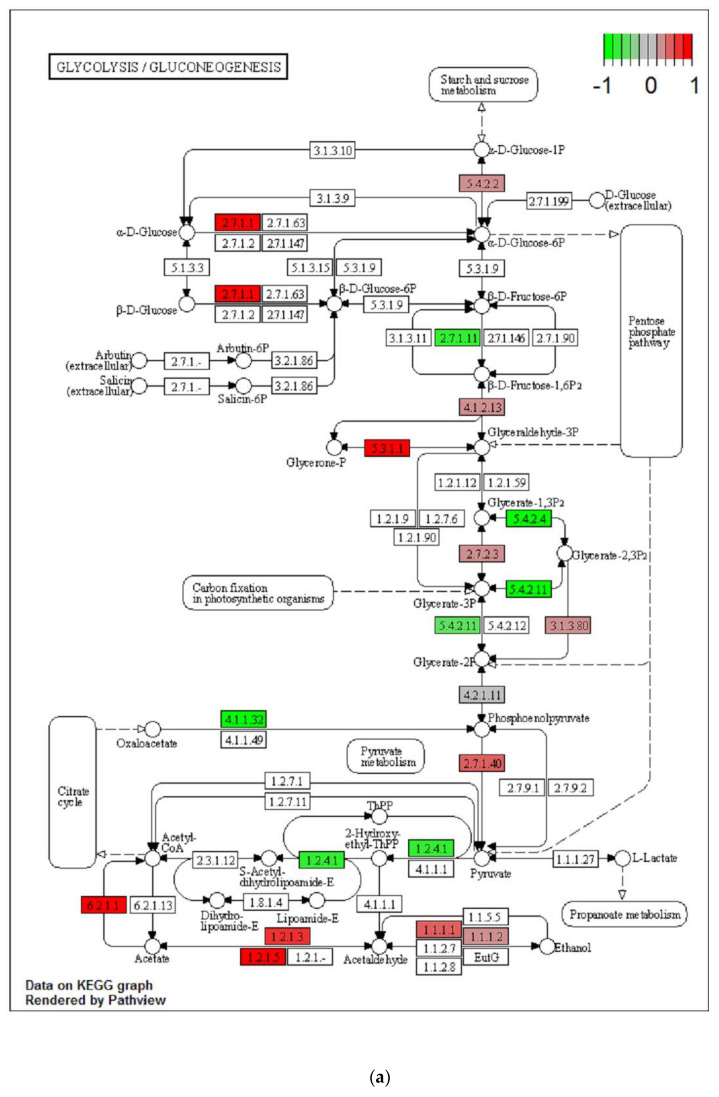

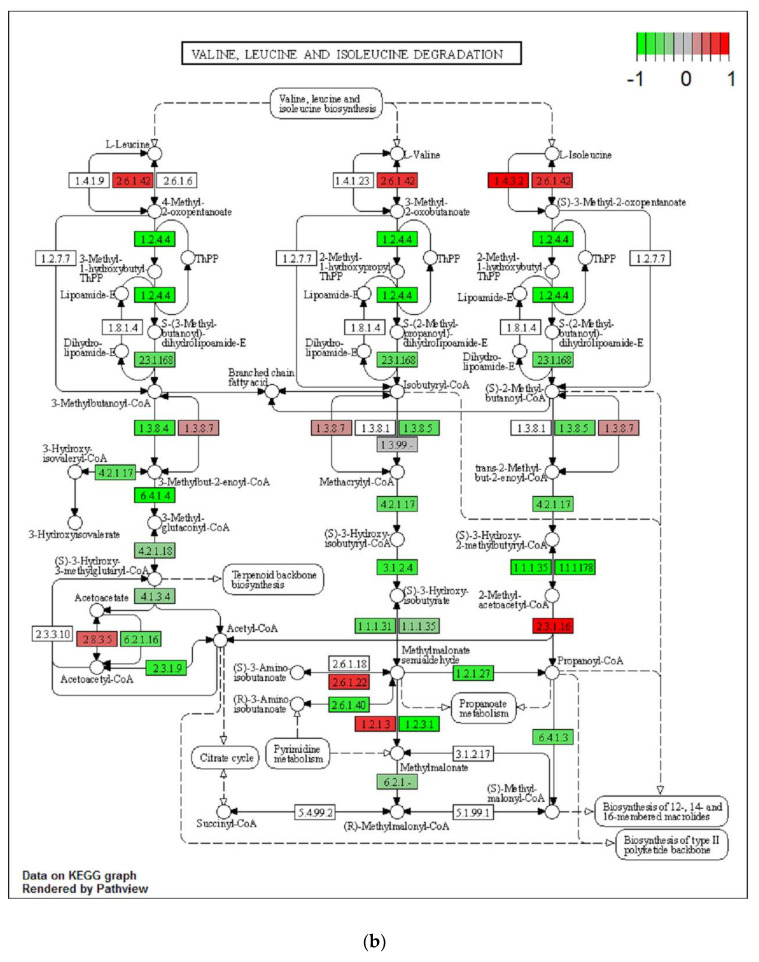

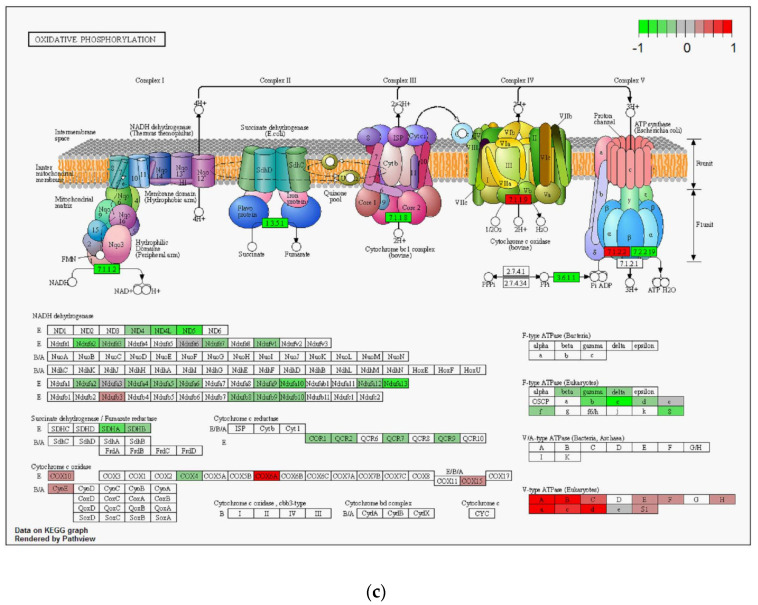
Regulation of genes related to glycolysis/neoglucogenesis (**a**), branched chain amino acid metabolism (**b**) and mitochondria (**c**) in HFoleic group compared to reference group. Down- and Up-regulations of transcript levels in VAT are represented in green and red, respectively. Regulations were mapped using Pathview (R-Bioconductor) and KEGG database.

We reported the expression ratio of corresponding genes on the schematic representation of BCAA degradation, glycolysis, and oxidative phosphorylation pathways. Expression ratios of non-mitochondrial related genes (from the list of significantly regulated genes after FDR correction) were also reported. Figure 5 shows the major effect in HFoleic compared to the reference group. By contrast only few genes were affected in HFepa group compared to the reference (Supplementary Figure S2).

### 2.5. Molecular Mediators of Differential Expression in VAT between Groups

Over-representation of known targets of mIR, transcription factors (TF), and TF complexes and families was identified among the lists of regulated transcripts identified at *p* < 0.05 after FDR correction in the HFoleic and HFepa groups compared to Ref group. We observed that 81 miRNA could be ubiquitously involved in the regulation of gene expression in both HF groups, but 34 were specifically over-represented in the comparison of the HFoleic and Ref groups. Furthermore, no TF exhibited a significant enrichment of targets to explain the differential expression values between the HFepa and Ref groups. On the contrary, several TF or complexes were identified as possible regulators of the adaptations in HFoleic group compared to the Ref group. HIF1a, SP1/3, STAT1/3, CEBPA/B, NFKB, and PPARg-RXR complexes exhibited a significant overrepresentation of targeted transcripts with a differential expression between the HFoleic and Ref groups (Table 2).

## 3. Discussion

Permanent modifications in molecular and physiological processes could occur during critical periods of prenatal growth. These changes may have long-term consequences and increase the susceptibility for developing obesity and its related metabolic diseases. It may be due to epigenetic mechanisms affecting gene expression or organ-specific structural and metabolic modifications, but the identification of the interaction of diet with all biological adaptations in the perinatal period is challenging [28]. It was demonstrated in a rodent model that a high intake of n-6 PUFA over four generations enhanced fat mass of the offspring [12]. Protection of offspring from weight gain, oxidative stress, and alteration in FA oxidation was demonstrated in animals with Fat1 overexpression that could increase the bioconversion of n-6 to n-3 PUFA [29]. We previously reported that transgenerational intake of EPA (provided by fish oil rich in EPA) could reduce body fat gain and cardiometabolic risk in offspring from the third generation when fed an obesogenic diet [23]. The current study aimed to investigate the specific transcriptomic adaptations in VAT during the development of obesity in these mice, which were compared to a control lineage that received an isocaloric supplementation with a high-oleic sunflower oil. We have previously reported the limitation of fibrotic, inflammatory, and anti-metabolic processes in the muscle and liver from these animals [23,24]. However, the biological and molecular adaptations induced by transgenerational intake of n-3 PUFA in the AT remained to be explored. Because of its major role in energy storage and as an endocrine organ, dysfunction in AT metabolism and secretory activity are crucial events in the alterations of physiological processes in obese subjects. They involve hypertrophy of adipocytes, increased inflammation, mitochondrial dysfunctions, fibrosis, and angiogenesis within the tissue [30,31,32]. Several lines of evidence have suggested that the intake of n-3 PUFA could protect from obesity and its related metabolic complications through the modification of adipose tissue secretory activity, inflammation, production of n-6 PUFA-derived eicosanoid and n-3 PUFA-derived protectins and resolvins [25,33]. The effects seems to depend on the metabolic status of the subjects and fat depots [34,35]. Visceral fat, which is more prone to immune cell infiltration, was found to be more responsive than subcutaneous fat [36,37], but the long term effects, involving the impact over several generations have never been investigated. Consequently, the current study was designed to identify transcriptomic adaptations in VAT to highlight the mechanisms contributing to the improved response to an obesogenic challenge in mice from the third generation of a lineage supplemented with 1% of n-3 PUFA (mainly EPA) in the diet.

We observed that transgenerational supplementation with EPA alleviated the obesogenic diet-induced disturbances in VAT lipid metabolism as it reduced the expression level of genes of the activation of cholesterol biosynthesis pathway. Some of the effects mediated by n-3 PUFA were proposed to be mediated by modifications at the level of adipokines expression/production. Our results are consistent with a differential expression of leptin induced by EPA intake. Leptin gene expression was significantly increased in the two lineages compared to Ref group (*p* ≤ 0.05 after FDR correction), but with a trend for a lower expression in HFepa compared to HFoleic group (*p* < 0.05 before FDR correction). No alteration of adiponectin gene expression was detected, although the expression of its receptors Adipor1 and Adipor2 was more reduced in obese mice from the HFoleic compared to the HFepa group. Our observations further support the hypotheses linking n-3 PUFA to the improvement in circulating leptin to adiponectin ratio [38].

Metabolic disorders induced by obesity were closely related to mitochondrial abnormalities such as an impairment in oxidative capacities and increased ROS production (see [39] for a recent review). Therefore, we performed a specific exploration of variation in mitochondria-related genes and observed that the obesogenic diet had a major impact on these genes. Despite a significant regulation in both the HFepa and HFoleic groups compared to the reference group, the impact of the obesogenic diet was markedly reduced in mice from the n-3 PUFA lineage (HFepa). The ontological analysis of the regulated genes showed that less pathways were represented and down-regulated in HFepa compared to the HFoleic group. It concerned the degradation of carbohydrates and fatty acids, but also the metabolism of amino acids. It was previously demonstrated that a high-fat diet with n-3 PUFA could increase energy expenditure in UCP1-deficient mice and be protective against obesity by increasing energy dissipation [40,41]. We also confirmed this effect in F3 mice from the EPA lineage receiving an obesogenic diet but no n-3 PUFA [23]. Almost all proteins involved in the BCAA degradation exhibited a significant regulation of their gene expression level in AT from HFoleic animals; although, only three were regulated in HFepa compared to Ref group. It has been found that perturbation of BCAA catabolism in the AT could lead to an increase in the circulating level of these amino acids, contributing to the development of insulin resistance [42]. Further studies are necessary to decipher how the metabolism of BCAA is affected in AT as both up and down regulation of genes from this pathway were observed in mice from the control lineage receiving the obesogenic diet compared to reference group fed with a normocaloric diet.

In direct line with their improvement of insulin resistance [23], HF-fed mice from the n-3 PUFA lineage exhibited a lowered alteration of mitochondrial-related processes and an increased expression of Irs2 in the AT compared to mice from the control lineage. Our previous study led to the hypothesis that a fine-tuning of IRS protein expression in the liver could contribute to the preservation of metabolic homeostasis, notably the regulation of hepatic glucose production in mice after EPA intake [24]. The present study further supports this hypothesis.

A major difference in transcriptomic adaptations between the EPA and control lineage concerned inflammation. N-3 PUFAs were described as reducing inflammatory events such as macrophage infiltration in the AT in both human and rodent models [21,35]. Such a beneficial effect was already observed in humans receiving long chain n-3 PUFA [43]. We also reported a decrease in the expression of *Foxo3* and *Gsk3beta* in the HFepa compared to the HFoleic group. We further showed using RT-qPCR, a significant down-regulation of Foxo3 expression in the HFepa group compared to the reference group. On the contrary *Akt1*, *Akt2*, and *Stat3* mRNA levels exhibited a reduced expression in HFepa compared to the HFoleic group. Foxo3 is one of the downstream effectors of chemokine signaling, and it could then be with NFKB, a key regulator of the nutrient-induced adaptation of gene expression. Hence, a specific enrichment in NFKB target genes was observed when HFoleic was compared to the reference/control group. The differential regulation of *Akt* and *Gsk3beta* between these two groups could be a key event in the metabolic adaption in VAT under an obesogenic environment, as it has been previously found that overexpression or increased activity of GSK3b protein was involved in insulin resistance and type 2 diabetes [44,45]. The adaptations in AT activity and inflammation with obesity are closely related to changes in the proportion of adipocytes, immune cells, endothelial cells, and stem cells which all possess specific biological roles [46,47]. We then compared the proportion of the different cell-types between the different groups and observed that it could not account for the differences in transcriptomic adaptations between the lineages. The observation of the lowered activation of inflammation was similar to observations in previous animal studies [21,22] where this effect was found to be independent of a reduction in fat mass. N-3 PUFAs could modulate AT response by inhibiting immune cells infiltration or activation, an interaction with PPAR transcription factors [48], or a direct effect on AT function [21]. The absence of apparent difference in the proportion of the different cell types in the VAT samples estimated from our transcriptomic study, argues in favor of a differential activation status. More recently, increased tissue accumulation of n-3 PUFA using the Fat1 overexpression model led to the prevention of weight gain during a high-fat diet which has been associated with specific persistent adaptations of the microbiota [49]; but unfortunately, we did not explore the variation in gut microbiota in our study.

## 4. Materials and Methods

### 4.1. Dietary Intervention and Sample Collection

**Figure 6:**
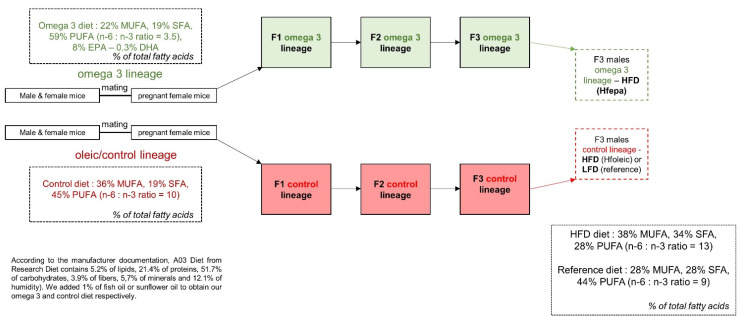
Design of the dietary intervention to obtain F3 animals from an n-3 PUFA and control lineage and explore the effect of a high-fat diet (HFD) in each lineage. MUFA, monounsaturated fatty acids; SFA, saturated fatty acids; PUFA, polyunsaturated fatty acids. HFD, high-fat diet; LFD, control low fat diet.

The present study was approved by the Animal Care and Use Committee of Auvergne (CEMEA Auvergne) and the Ministère de l’Enseignement Supérieur et de la Recherche (01276.01). C57bl/6J mice were bred for 3 generations and housed in the animal facility the INRAE research center of Theix (Saint-Genes Champanelle, France) with a growing diet (A03 diet from Safe diets, Augy, France) supplemented with 1% (w:w) of fish oil (Omegavie® EPA, 70 TG, Polaris, Quimper, France) containing 75% of n-3 PUFA (omega 3 lineage), mainly in the form of EPA which represented 8% of the fatty acids in the final diet or 1% (w:w) of high-oleic sunflower oil (control lineage) containing 83.5% of oleic acid (Lesieur, Coudekerque Branche, France) as a control condition. The supplementation of the control group with 1% of sunflower oil aimed at matched the caloric supplementation related to the addition of fish oil in the omega 3 lineage. The details of the breeding strategy and lipid composition of the diets were described previously [23] and are summarized in Figure 6. Eight F3 male mice from oleic/control (HFoleic) and EPA/omega 3 (HFepa) lineages were matched for body weight (*n* = 8 per group) and fed a high-fat, high-sucrose diet (HFD, 24% of fat, 20% of sucrose) providing 45% of energy from fat (RD 12,451 from Research diet, Brogaarden Gentofte, Denmark) for 17 weeks. These animals constituted the HFoleic and HFepa groups, respectively. A reference group of F3 male mice (*n* = 8) from the control lineage was fed with a low-fat reference diet (Ref) providing 10% of energy from fat (RD 12450H from Research diet) during the challenge as a reference group. Animals were maintained under a temperature-controlled environment and 12 h–12 h light-dark cycle throughout the study. All the procedures were followed to reduce the number and manipulation of the animals in the study.

At the end of the feeding period, during the fasting period, the animals were sacrificed under anesthesia with 4% isoflurane. Visceral adipose tissue (VAT) from the epididymal area was harvested, snap-frozen in liquid nitrogen, and stored at −80 °C until use. Total RNA was extracted using TRIzol^®^ reagent (Thermo Fisher Scientific, Waltham, MA, USA) according to the manufacturer’s instructions. Each total RNA sample was assessed for quantification and integrity using the Agilent Bioanalyzer (Agilent, Santa Clara, CA USA). Only RNA samples exhibiting a RIN index > 6.5 were used in the current study (Ref, *n* = 6; HFoleic, *n* = 6; HFepa, *n* = 8) for microarray experiments.

### 4.2. Transcriptomic Analysis

Gene expression profiles were performed at the GeT-TRiX facility (GénoToul, Génopole Toulouse Midi-Pyrénées) using Agilent Sureprint G3 Mouse GE v2 microarrays (8 × 60 K, design 074809) following the manufacturer’s instructions. For each sample, Cyanine-3 (Cy3) labeled cRNA was prepared from 200 ng of total RNA using the One-Color Quick Amp Labeling kit (Agilent Technologies) according to the manufacturer’s instructions, followed by Agencourt RNAClean XP (Agencourt Bioscience Corporation, Beverly, Massachusetts). Dye incorporation and cRNA yield were checked using Dropsense™96 UV/VIS droplet reader (Trinean NV, Gent, Belgium). 600 ng of Cy3-labelled cRNA were hybridized on the microarray slides following the manufacturer’s instructions. Immediately after washing, the slides were scanned on Agilent G2505C Microarray Scanner using Agilent Scan Control A.8.5.1 software and fluorescence signal extracted using Agilent Feature Extraction software v10.10.1.1 with default parameters. Microarray data and experimental details are available in NCBI’s Gene Expression Omnibus [50] and are accessible through GEO Series accession number GSE176356.

### 4.3. RT-q PCR Validation of Gene Expression Change

Total RNA was extracted from VAT muscle using TRIzol^®^ reagent Thermo Fisher Scientific, Waltham, MA, USA) according to the manufacturer’s instructions. Each total RNA sample was assessed for quantification and integrity using the Agilent Bioanalyzer (Agilent, Santa Clara, CA, USA). cDNAs were synthesized from 2 µg of total RNA using the High-Capacity cDNA Reverse Transcription Kit from Applied Biosystem (Thermo Fisher Scientific, Waltham, MA, USA). The products of reverse transcription were used for Quantitative Real Time Polymerase Chain Reaction (qRT-PCR) using specific primers and Rotor-Gene SYBR Green PCR master mix on a Rotor-Gene Q system (Qiagen, Courtaboeuf, France). Messenger RNA (mRNA) quantification was assayed using the ddCT method using hprt as internal housekeeping gene. Primer sequences and PCR conditions are available upon request (frederic.capel@inrae.fr).

### 4.4. Statistics

Microarray data were analyzed using R [51] and Bioconductor packages (www.bioconductor.org, v 3.6, accessed on September 2018, [52]). Raw data (median signal intensity) were filtered, and log2 transformed and normalized using quantile method [53]. A model was fitted using the limma lmFit function [54]. Pair-wise comparisons between biological conditions were applied using specific contrasts and a correction for multiple testing using Benjamini–Hochberg procedure [55] to control the False Discovery Rate (FDR). Probes with FDR-adjusted *p* < 0.05 were considered to be differentially expressed between conditions, excepted when GSEA was used. In order to verify the classification group (condition) of our samples, we performed a partial least squares regression–discriminant analysis (PLS-DA). PLS-DA was performed from differentially expressed genes and miRNA using Mixomics in R [56]. In addition, hierarchical clustering of samples and gene expression data was performed using Euclidean distance and Ward’s method using R-Bioconductor libraries.

RT-qPCR data were analyzed using pairwise t-tests and a significance threshold fixed at *p* < 0.05.

### 4.5. Characterization of Cell-Type Abundance

Cibersort [26] was used to characterize the heterogeneity in VAT cellular populations according to a previously identified signature [57].

### 4.6. Biological Pathway Analyses

Molecular signatures in gene lists were explored using GeneTrail2 [58] and GSEA to identify significant enrichment in biological pathways, processes. The identification of potential transcription factors (TF), and TF families or complexes involved in gene expression changes was performed using an over-representation analysis in GeneTrail2 following the default statistical recommendations.

The CIBERSORT software was used to calculate the abundance of the different cell-types composing adipose tissue based on the dataset establish by Lenz et al. [26,57]. The identification of genes related to mitochondria was performed using the MitoMiner online database [27]. Analysis of gene ontology of mitochondria targeted genes was performed using the David database [59,60]. Significant regulations were mapped on pathways according to the KEGG (Kyoto Encyclopedia of Genes and Genomes) database using the Pathview package [61] from Bioconductor implemented in R.

## 5. Conclusions

The present study identified a differential adaptation of the VAT transcriptome, which could contribute to a better metabolic response in mice deriving from a lineage receiving supplementation with n-3 PUFA under an obesogenic stress. Supplementation of three generations of mice with 1% of fish oil containing 75% of EPA induced an improvement in inflammation, the use of the different energetic substrates and mitochondrial functions, lipid homeostasis, and insulin response in the offspring exposed to an obesogenic diet.

## Figures and Tables

**Table 1:** Comparison of VAT’s gene expression data in microarray and RT-qPCR measurements. Values are mRNA level ratio between the different groups (*n* = 6/8 per group). Microarray experiments identified significant differential expression with *p* < 0.05 with FDR correction (*) or no FDR correction (a). RT-qPCR validation experiments identified significant differential expression with *p* < 0.05 (b) or *p* < 0.1 (c). HFoleic, mice from oleic/control lineage fed with the obesogenic diet; HFepa, mice from EPA/omega 3 lineage fed with the obesogenic diet; Ref, mice from oleic/control lineage fed with the normocaloric diet.

		Microarray			RT-qPCR	
Genes	HFoleic vs. Ref	HFepa vs. HFoleic	HFepa vs. Ref	HFoleic vs. Ref	HFepa vs. HFoleic	HFepa vs. Ref
*Dnajb1*	1.00	1.20 a	1.20a	0.93	1.12 b	1.05
*Dock8*	4.38 *	0.81	3.53 *	3.56 b	0.82 c	2.92 b
*Foxo3*	1.10	0.86 a	0.95	0.91	0.85 c	0.77 b
*Irs2*	0.51 *	1.54 a	0.79	0.49 b	1.38 b	0.68 b
*Leptin*	2.18 *	0.74 a	1.62	4.97 a	0.46 a	2.30
*Mmp13*	1.80 *	0.63 a	1.13	4.40 b	0.38 b	1.66
*Nupl2*	0.96	0.83 a	0.79 *	0.86 c	0.82 b	0.71 b
*Plin2*	1.94 *	0.76	1.47 a	1.65 b	0.66 b	1.09
*Serpine1*	0.38 *	2.02 a	0.77	0.37 b	1.94 c	0.72
*Stat3*	0.78 *	1.13	0.88	0.63 b	0.96	0.60 b
*Timp3*	0.61 *	1.45 a	0.88	0.59 b	1.18	0.70 b

**Table 2:** Significant overrepresentation in VAT of validated targets of transcription factors (TF), TF complexes or families in HFoleic vs. Reference group comparison.

	Name	Hits (*n*)	Adjusted *p*-Value
Transcription factors	SP1	104	<0.001
	SP3	45	<0.001
	SPI1	27	<0.001
	CEBPB	31	<0.001
	IRF1	16	<0.01
	IRF8	12	<0.01
	HIF1A	16	<0.01
	STAT1	15	<0.01
	YY1	18	<0.01
	USF1	15	<0.01
	CEBPA	17	<0.01
	EGR1	15	<0.01
	RELA	15	<0.05
	ETS1	12	<0.05
	POU2F1	14	<0.05
	STAT3	13	<0.05
	NFYA	12	<0.05
Transcription factor complexes	AP-1	24	<0.001
	NF-kappaB	14	<0.05
	PPARgamma:RXR-alpha	8	<0.05
	RelA-p65:p50	9	<0.05
	USF2:usf1	8	<0.05
Transcription factor families	NF-1	14	<0.05

## Data Availability

Microarray data and experimental details are accessible through GEO Series accession number GSE176356.

## Supplementary Materials

The following are available online at www.mdpi.com/article/10.3390/metabo11120838/s1, Figure S1: Differential regulation of Chemokine signaling pathway between the HFepa and HFoleic groups, Figure S2: Alteration of mitochondria-targeted genes in the HFepa vs. Ref groups, Table S1: GSEA identified regulations in processes and pathways using transcriptomic data when comparing the HFepa and HFoleic groups, Table S2: Differential regulation of mitochondria targeted genes between the HFoleic and HFepa groups compared to the Ref group, Table S3: Enriched pathways among mitochondria-targeted genes.
