# Supplementary material for: Adipose Tissue Dysfunctions in Response to an Obesogenic Diet Are Reduced in Mice after Transgenerational Supplementation with Omega 3 Fatty Acids
Source: Metabolites. 2021 Dec 4;11(12):838. doi: 10.3390/metabo11120838 (PMC8706165; doi:10.3390/metabo11120838)
Supplement: Supplementary file 1 [file metabolites-11-00838-s001.zip › suppl table S1.pdf]

Supplementary Table S1. HFepa vs HFoleic comparison using GSEA identified enriched gene sets

#Name	Reference	Hits	*P*-val	HFepa vs HFoleic
mRNA processing	WIKI	149	1.43E-08	enriched
Chemokine signaling pathway	WIKI	64	3.15E-03	depleted
Cholesterol Biosynthesis	WIKI	11	3.15E-03	depleted
DNA Replication	WIKI	23	3.15E-03	depleted
GPCRs, Class A Rhodopsin-like	WIKI	37	3.15E-03	depleted
Spinal Cord Injury	WIKI	27	3.15E-03	depleted
Non-odorant GPCRs	WIKI	58	4.79E-03	depleted
IL-5 Signaling Pathway	WIKI	29	6.67E-03	depleted
GPCRs, Class B Secretin-like	WIKI	9	1.94E-02	enriched
Matrix Metalloproteinases	WIKI	11	1.94E-02	depleted
Macrophage markers	WIKI	6	4.29E-02	depleted
Mitotic Prometaphase	Reactome	50	7.08E-07	depleted
Resolution of Sister Chromatid Cohesion	Reactome	54	7.08E-07	depleted
RHO GTPases Activate Formins	Reactome	58	9.62E-07	depleted
Separation of Sister Chromatids	Reactome	78	4.84E-06	depleted
Collagen degradation	Reactome	20	8.89E-06	depleted
Immunoregulatory interactions between a Lymphoid and a non-Lymphoid cell	Reactome	34	1.07E-05	depleted
Assembly of collagen fibrils and other multimeric structures	Reactome	18	9.07E-05	depleted
Degradation of the extracellular matrix	Reactome	19	9.07E-05	depleted
NCAM1 interactions	Reactome	8	9.07E-05	depleted
MHC class II antigen presentation	Reactome	39	1.56E-04	depleted
Meiotic Recombination	Reactome	25	1.60E-04	depleted
RHO GTPases Activate NADPH Oxidases	Reactome	5	1.60E-04	depleted
Integrin cell surface interactions	Reactome	33	9.21E-04	depleted
Regulation of actin dynamics for phagocytic cup formation	Reactome	23	1.29E-03	depleted
Collagen biosynthesis and modifying enzymes	Reactome	24	1.96E-03	depleted
G alpha (s) signalling events	Reactome	27	3.36E-03	enriched
Extracellular matrix organization	Reactome	7	4.68E-03	depleted
Non-integrin membrane-ECM interactions	Reactome	7	4.68E-03	depleted
Cell surface interactions at the vascular wall	Reactome	22	5.00E-03	depleted
ECM proteoglycans	Reactome	18	7.07E-03	depleted

GPVI-mediated activation cascade	Reactome	20	7.07E-03	depleted
Signaling by PDGF	Reactome	10	1.01E-02	depleted
mRNA Splicing - Major Pathway	Reactome	37	1.06E-02	enriched
G alpha (i) signalling events	Reactome	32	1.23E-02	depleted
Gamma-carboxylation of protein precursors	Reactome	2	1.23E-02	depleted
Removal of aminoterminal propeptides from gamma-carboxylated proteins	Reactome	2	1.23E-02	depleted
Transport of gamma-carboxylated protein precursors from the endoplasmic reticulum to the Golgi apparatus	Reactome	2	1.23E-02	depleted
Cholesterol biosynthesis	Reactome	12	3.01E-02	depleted
Cyclin A/B1 associated events during G2/M transition	Reactome	9	3.01E-02	depleted
Beta defensins	Reactome	4	3.37E-02	depleted
Phagosome	KEGG	67	2.87E-11	depleted
Staphylococcus aureus infection	KEGG	23	3.05E-09	depleted
Lysosome	KEGG	60	8.85E-09	depleted
Tuberculosis	KEGG	64	3.82E-07	depleted
Osteoclast differentiation	KEGG	56	2.22E-06	depleted
Systemic lupus erythematosus	KEGG	38	3.60E-06	depleted
Allograft rejection	KEGG	18	1.94E-05	depleted
Leishmaniasis	KEGG	26	1.94E-05	depleted
Antigen processing and presentation	KEGG	34	4.82E-05	depleted
Cell adhesion molecules (CAMs)	KEGG	53	4.82E-05	depleted
Graft-versus-host disease	KEGG	20	4.82E-05	depleted
Natural killer cell mediated cytotoxicity	KEGG	50	5.18E-05	depleted
Autoimmune thyroid disease	KEGG	19	9.65E-05	depleted
Rheumatoid arthritis	KEGG	33	1.26E-04	depleted
Protein digestion and absorption	KEGG	27	1.45E-04	depleted
B cell receptor signaling pathway	KEGG	33	1.60E-04	depleted
Viral myocarditis	KEGG	27	1.66E-04	depleted
Type I diabetes mellitus	KEGG	18	1.68E-04	depleted
Asthma	KEGG	8	2.72E-04	depleted
Amoebiasis	KEGG	39	2.76E-04	depleted
Cytokine-cytokine receptor interaction	KEGG	66	4.30E-04	depleted
Spliceosome	KEGG	44	7.96E-04	enriched
Chemokine signaling pathway	KEGG	62	1.25E-03	depleted
Hematopoietic cell lineage	KEGG	27	1.31E-03	depleted
Cell cycle	KEGG	52	1.68E-03	depleted

Steroid biosynthesis	KEGG	12	3.84E-03	depleted
Fc gamma R-mediated phagocytosis	KEGG	36	3.88E-03	depleted
DNA replication	KEGG	23	6.59E-03	depleted
ECM-receptor interaction	KEGG	33	6.59E-03	depleted
Viral carcinogenesis	KEGG	76	8.75E-03	depleted
Intestinal immune network for IgA production	KEGG	17	9.11E-03	depleted
Malaria	KEGG	15	2.83E-02	depleted
Toll-like receptor signaling pathway	KEGG	43	2.83E-02	depleted
Biosynthesis of amino acids	KEGG	22	2.95E-02	depleted
Oocyte meiosis	KEGG	36	3.81E-02	depleted
Herpes simplex infection	KEGG	65	4.56E-02	depleted
Eicosanoid Metabolism	Biocarta	9	7.9 E-03	depleted
P-val= *p* value				

Gene Set Enrichment Analysis from GeneTrail was used to compare HFoleic and HFepa groups and identified differentially regulated biological processes, pathways, reactions or molecular functions. P values were adjusted for multiple testing using the Benjamini and Yekutieli's method, as recommended by GeneTrial.
