# Supplementary material for: Adipose Tissue Dysfunctions in Response to an Obesogenic Diet Are Reduced in Mice after Transgenerational Supplementation with Omega 3 Fatty Acids
Source: Metabolites. 2021 Dec 4;11(12):838. doi: 10.3390/metabo11120838 (PMC8706165; doi:10.3390/metabo11120838)
Supplement: Supplementary file 1 [file metabolites-11-00838-s001.zip › suppl table S3.pdf]

up-regulated KEGG pathways in HFoleic group vs reference	Count	% involved genes	Pvalue (FDR)	Genes
mmu01130:Biosynthesis of antibiotics	29	9.2	8.22E-13	ACAA2, SHMT2, ENO1B, ACAA1A, HK1, GCSH, HK3, ALDH1B1, PPAT, PGK1, ACADM, ACSS1, FDPS, TPI1, ARG1, IDH1, NME2, PYCR1, NME4, LSS, ASS1, NME1, ALDH3A2, HADHB, SUCLA2, PKM, BCAT1, ALDOA, ALDH9A1
mmu01230:Biosynthesis of amino acids	12	3.8	3.57E-05	TPI1, PKM, SHMT2, ARG1, IDH1, ENO1B, PGK1, PYCR1, ALDH18A1, BCAT1, ALDOA, ASS1
mmu00071:Fatty acid degradation	10	3.2	3.64E-05	ALDH3A2, HADHB, CPT1A, ACAA2, ALDH1B1, ACSL5, ACADM, ACAA1A, ACOX3, ALDH9A1
mmu00010:Glycolysis / Gluconeogenesis	11	3.5	4.56E-05	ALDH3A2, HK3, TPI1, PKM, ALDH1B1, ENO1B, PGK1, ACSS1, ALDOA, ALDH9A1, HK1
mmu00670:One carbon pool by folate	7	2.2	5.75E-05	DHFR, ALDH1L1, MTHFD1L, SHMT2, MTHFD2, MTHFSL, MTHFS
mmu00280:Valine, leucine and isoleucine degradation	10	3.2	5.75E-05	ALDH3A2, HADHB, ACAA2, ALDH1B1, OXCT1, ABAT, ACADM, ACAA1A, BCAT1, ALDH9A1
mmu04141:Protein processing in endoplasmic reticulum	16	5.1	7.15E-05	PDIA3, HSPA8, SSR4, HSPA5, PRKCSH, TUSC3, RPN1, MOGS, RRBP1, DNAJA1, LMAN1, BAX, BAK1, P4HB, CALR, CRYAB
mmu00330:Arginine and proline metabolism	9	2.9	1.68E-04	ALDH3A2, GATM, P4HA1, ALDH1B1, ARG1, MAOA, PYCR1, ALDH18A1, ALDH9A1
mmu01212:Fatty acid metabolism	9	2.9	2.06E-04	HADHB, CPT1A, ACAA2, OXSM, TECR, ACSL5, ACADM, ACAA1A, ACOX3
mmu01200:Carbon metabolism	12	3.8	6.28E-04	HK3, SUCLA2, TPI1, PKM, SHMT2, IDH1, ENO1B, PGK1, ACADM, ACSS1, ALDOA, HK1
mmu03010:Ribosome	12	3.8	4.29E-03	RPL4, MRPL20, RPL30, RPS16, RPL10, MRPL18, MRPS11, MRPL19, MRPL27, RPL36A, MRPL34, MRPS6
mmu04146:Peroxisome	9	2.9	5.42E-03	PRDX5, IDH1, PRDX1, AGPS, HSD17B4, ACSL5, ACAA1A, ACOX3, ABCD1
mmu00051:Fructose and mannose metabolism	6	1.9	9.14E-03	GMPPB, HK3, TPI1, AKR1B3, ALDOA, HK1
mmu04142:Lysosome	10	3.2	0.0147470 67	CTSA, SCARB2, ASAH1, GM2A, PSAP, CLTC, TPP1, TCIRG1, CTSD, CTSB
mmu00620:Pyruvate metabolism	6	1.9	0.0151583 68	ALDH3A2, PKM, ALDH1B1, ACYP2, ACSS1, ALDH9A1

down-regulated KEGG pathways in HFoleic group vs reference	Count	% involved genes	Pvalue (FDR)	Genes
mmu00190:Oxidative phosphorylation	35	9.4	2.48E-24	NDUFB9, NDUFB8, NDUFA13, UQCRB, NDUFB10, NDUFA12, COX4I1, NDUFA10, ATP5K, ATP5C1, ATP5G3, UQCR10, ATP5H, ATP5G2, ATP5G1, ATP5B, ATP5D, NDUFV1, NDUFA9, NDUFA6, NDUFA5, NDUFA4, NDUFA3, NDUFA2, SDHA, ATP5F1, SDHB, ATP5J2, NDUFS7, PPA2, NDUFS6, UQCRC1, NDUFS3, NDUFS2, UQCRC2
mmu05012:Parkinson's disease	33	8.8	4.28E-21	NDUFB9, NDUFB8, NDUFA13, UQCRB, NDUFB10, NDUFA12, COX4I1, NDUFA10, ATP5C1, ATP5G3, PARK7, UQCR10, ATP5H, ATP5G2, ATP5G1, ATP5B, ATP5D, NDUFV1, NDUFA9, NDUFA6, NDUFA5, NDUFA4, NDUFA3, NDUFA2, SDHA, ATP5F1, SDHB, NDUFS7, NDUFS6, UQCRC1, NDUFS3, NDUFS2, UQCRC2
mmu00280:Valine, leucine and isoleucine degradation	22	5.9	1.90E-19	ACAD8, BCKDHA, MCCC2, ECHS1, HIBADH, MCCC1, BCKDHB, ACSF3, HSD17B10, ACADSB, ACAT1, HMGCL, ALDH6A1, ALDH2, AUH, IVD, DBT, PCCB, HADH, ALDH7A1, HIBCH, BCAT2

mmu05010:Alzheimer's disease	33	8.8	6.72E-19	NDUFB9, NDUFB8, NDUFA13, UQCRB, NDUFB10, NDUFA12, COX4I1, NDUFA10, ATP5C1, ATP5G3, UQCR10, ATP5H, ATP5G2, HSD17B10, ATP5G1, ATP5B, ATP5D, NDUFV1, NDUFA9, NDUFA6, NDUFA5, NDUFA4, NDUFA3, NDUFA2, SDHA, ATP5F1, SDHB, NDUFS7, NDUFS6, UQCRC1, NDUFS3, NDUFS2, UQCRC2
mmu05016:Huntington's disease	33	8.8	1.88E-17	NDUFB9, NDUFB8, NDUFA13, UQCRB, NDUFB10, NDUFA12, COX4I1, NDUFA10, ATP5C1, ATP5G3, UQCR10, ATP5H, ATP5G2, ATP5G1, ATP5B, ATP5D, NDUFV1, NDUFA9, NDUFA6, NDUFA5, NDUFA4, NDUFA3, NDUFA2, SDHA, ATP5F1, SDHB, SOD1, NDUFS7, NDUFS6, UQCRC1, NDUFS3, NDUFS2, UQCRC2
mmu01130:Biosynthesis of antibiotics	30	8.0	1.16E-13	ECHS1, DLST, AK4, PDHB, HSD17B10, PAPSS2, ACAT1, ATIC, ALDH2, DBT, IDH3B, HADH, PRODH, PCK2, BCKDHA, PDHA1, MDH1, IDH3G, BCKDHB, NME3, SDHA, SDHB, CAT, PCCB, OGDH, SUCLG2, SUCLG1, ACO2, ALDH7A1, BCAT2
mmu00020:Citrate cycle (TCA cycle)	14	3.7	1.41E-12	PDHA1, PCX, MDH1, IDH3G, DLST, PDHB, SDHA, SDHB, OGDH, IDH3B, SUCLG2, SUCLG1, ACO2, PCK2
mmu04932:Non-alcoholic fatty liver disease (NAFLD)	24	6.4	1.38E-11	NDUFA9, NDUFB9, NDUFB8, NDUFA13, NDUFA6, NDUFA5, UQCRB, NDUFB10, NDUFA4, NDUFA12, NDUFA3, COX4I1, NDUFA2, NDUFA10, UQCR10, SDHA, SDHB, NDUFS7, NDUFS6, UQCRC1, NDUFS3, NDUFS2, UQCRC2, NDUFV1
mmu01200:Carbon metabolism	20	5.3	1.79E-10	PDHA1, ECHS1, PCX, MDH1, IDH3G, GPT2, DLST, PDHB, SDHA, SDHB, ACAT1, ALDH6A1, CAT, PCCB, OGDH, IDH3B, SUCLG2, SUCLG1, ACO2, HIBCH
mmu00640:Propanoate metabolism	10	2.7	6.26E-08	ACSS3, ALDH6A1, ECHS1, PCCB, SUCLG2, SUCLG1, MLYCD, ACACB, HIBCH, ACAT1
mmu00620:Pyruvate metabolism	11	2.9	1.37E-07	GRHPR, PDHA1, PCX, ALDH2, MDH1, LDHD, PDHB, ACACB, ALDH7A1, PCK2, ACAT1
mmu00071:Fatty acid degradation	9	2.4	1.46E-04	ACADVL, ECHS1, ALDH2, ACSL1, ECI1, HADH, ACADSB, ALDH7A1, ACAT1
mmu01212:Fatty acid metabolism	9	2.4	1.85E-04	PECR, ACADVL, ECHS1, ACSL1, HADH, ACADSB, MCAT, FADS1, ACAT1
mmu00650:Butanoate metabolism	7	1.9	2.51E-04	HMGCL, ECHS1, ACSM3, ACSM5, L2HGDH, HADH, ACAT1
mmu00630:Glyoxylate and dicarboxylate metabolism	7	1.9	3.64E-04	GRHPR, MDH1, CAT, PCCB, ACO2, GLUL, ACAT1
mmu00380:Tryptophan metabolism	8	2.1	7.22E-04	ECHS1, ALDH2, CAT, KYAT3, OGDH, HADH, ALDH7A1, ACAT1
mmu04146:Peroxisome	10	2.6	8.80E-04	PECR, HMGCL, PHYH, SCP2, ACSL1, PXMP2, CAT, MLYCD, CRAT, SOD1
mmu00310:Lysine degradation	8	2.1	1.25E-03	ECHS1, ALDH2, PHYKPL, OGDH, DLST, HADH, ALDH7A1, ACAT1
mmu03010:Ribosome	12	3.2	3.48E-03	MRPL30, MRPS15, MRPS9, RPL36, MRPS18A, MRPL9, MRPL13, RPL6, RPS13, MRPL32, MRPS5, RPL7
mmu01210:2-Oxocarboxylic acid metabolism	5	1.3	5.26E-03	IDH3G, GPT2, IDH3B, ACO2, BCAT2
mmu00410:beta-Alanine metabolism	6	1.6	5.40E-03	ALDH6A1, ECHS1, ALDH2, MLYCD, ALDH7A1, HIBCH
mmu00260:Glycine, serine and threonine metabolism	6	1.6	0.012573089	GRHPR, ALAS2, AGXT2, CHDH, ALDH7A1, GCAT
mmu00860:Porphyrin and chlorophyll metabolism	6	1.6	0.013469612	ALAS2, FECH, CPOX, PPOX, EPRS, FXN

mmu01230:Biosynthesis of amino acids	7	1.9	0.043709208	PCX, IDH3G, GPT2, IDH3B, ACO2, GLUL, BCAT2
mmu00480:Glutathione metabolism	6	1.6	0.044522901	GPX1, GSTA4, GSTP1, MGST3, GSR, MGST1
**down-regulated KEGG pathways in HFepa group vs reference**	**Count**	**% involved genes**	**Pvalue (FDR)**	**Genes**
mmu00280:Valine, leucine and isoleucine degradation	5	11.9	0.000622773	MCCC2, ALDH2, IVD, MCCC1, ACADSB

Enrichment in cellular pathways from the KEGG database in mitochondria-targeted genes identified for: HFoleic group vs reference and HFepa group vs reference comparisons. Identification and statistical analysis were performed using the DAVID online tool.

Up regulated pathways are shown in red. Down regulated pathways are shown in green. No significant up regulated pathways were identified when HFepa and reference groups were compared.
