# Supplementary figures and images for: Adipose Tissue Dysfunctions in Response to an Obesogenic Diet Are Reduced in Mice after Transgenerational Supplementation with Omega 3 Fatty Acids
Source: Metabolites. 2021 Dec 4;11(12):838. doi: 10.3390/metabo11120838 (PMC8706165; doi:10.3390/metabo11120838)

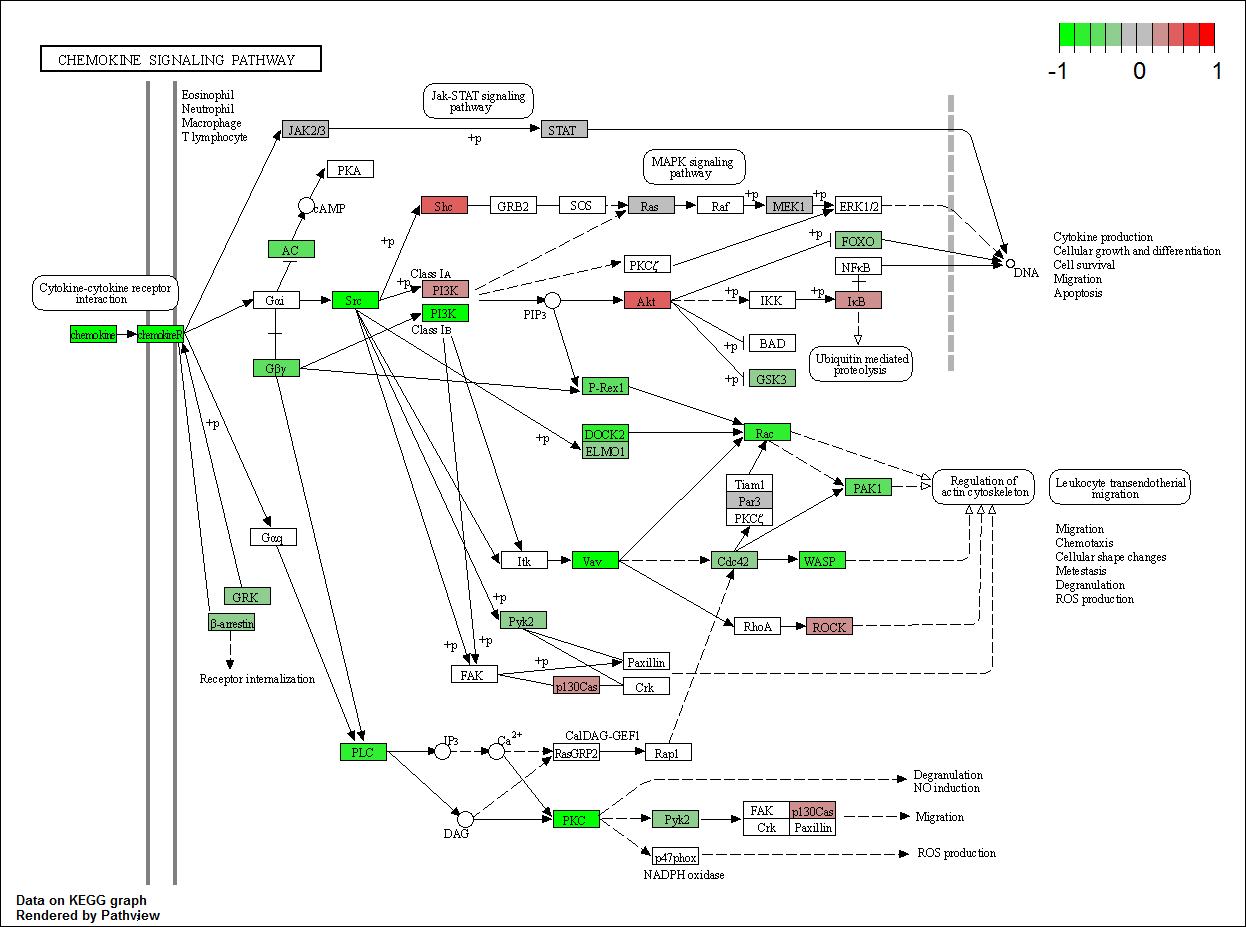

Supplement: Supplementary file 1 [file metabolites-11-00838-s001.zip › FigS1.png]

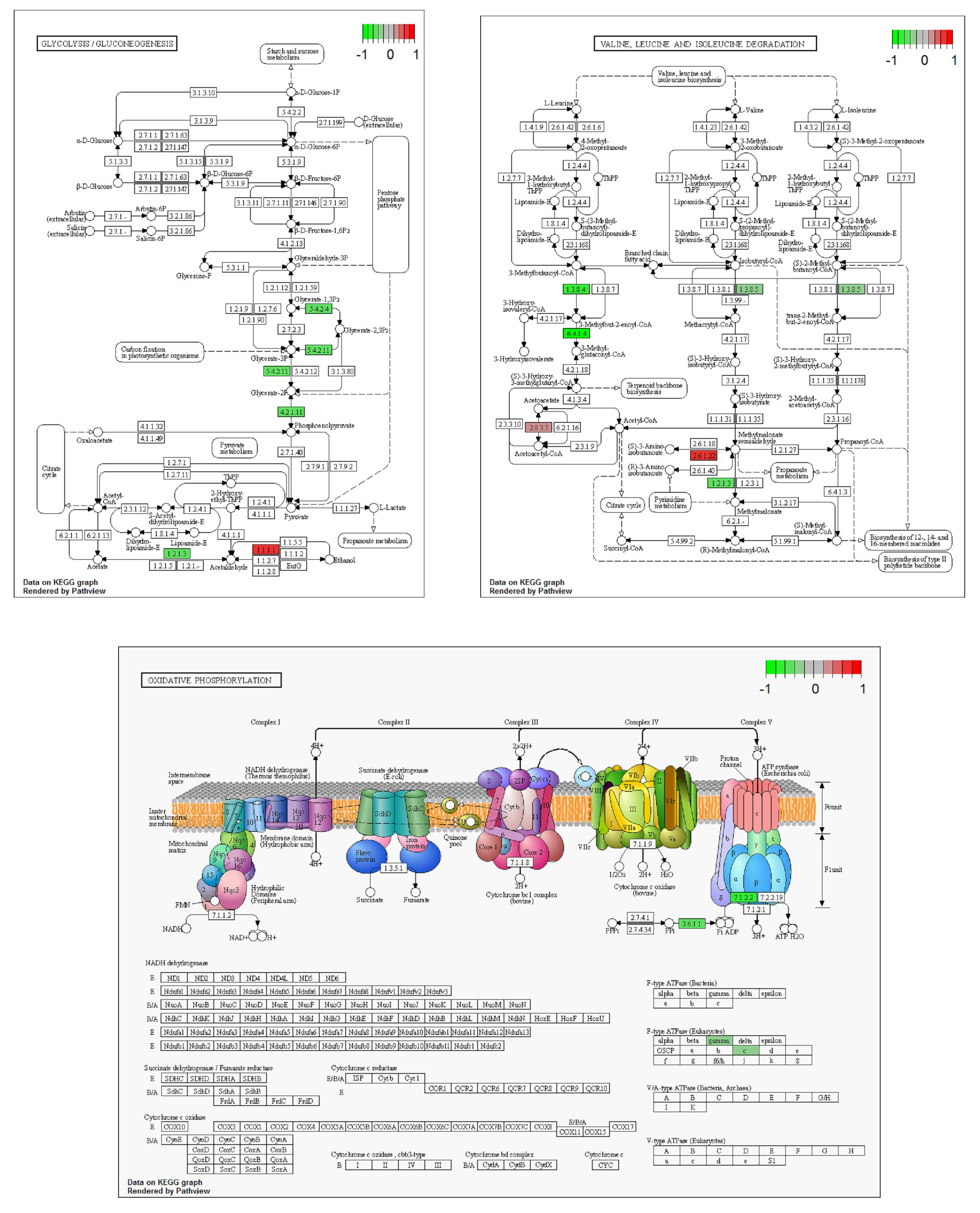

Supplement: Supplementary file 1 [file metabolites-11-00838-s001.zip › FigS2.jpg]
